# The Heavy Links between Geological Events and Vascular Plants Evolution: A Brief Outline

**DOI:** 10.1155/2016/9264357

**Published:** 2016-02-04

**Authors:** Aldo Piombino

**Affiliations:** Earth Sciences Department, Florence University, 50125 Florence, Italy

## Abstract

Since the rise of photosynthesis, life has influenced terrestrial atmosphere, particularly the O_2_ and the CO_2_ content (the latter being originally more than 95%), changing the chemistry of waters, atmosphere, and soils. Billions of years after, a far offspring of these first unicellular forms conquered emerging lands, not only completely changing landscape, but also modifying geological cycles of deposition and erosion, many chemical and physical characteristics of soils and fresh waters, and, more, the cycle of various elements. So, there are no doubts that vascular plants modified geology; but it is true that also geology has affected (and, more, has driven) plant evolution. New software,* PyRate*, has determined vascular plant origin and diversification through a Bayesian analysis of fossil record from Silurian to today, particularly observing their origination and extinction rate. A comparison between* PyRate* data and geological history suggests that geological events massively influenced plant evolution and that also the rise of nonflowering seed plants and the fast diffusion of flowering plants can be explained, almost partly, with the environmental condition changes induced by geological phenomena.

## 1. Natural Drivers of Climate Changes and Their Effects on Evolution

Climate is regulated firstly by the amount of solar radiation reaching the surface; this value is mainly a function of the latitude. But there are other astronomical factors regulating it. Earth orbit variations are globally felt: the effects of orbital forcing, known as* Milankovitch cycles*, have been well recognized in many sedimentary series in plenty of geochemical and stratigraphic parameters; there are also changes in solar activity rate at a pluricentennial scale: for example, in Holocene colder and warmer period (such as the* Medieval climatic optimum* and the* Little Ice Age*) are linked to these variations that can produce shifts of the limits of climatic belts. All these changes are poorly reflected in life evolution because they are too brief to promote evolution. From upper Tertiary the onset of the Antarctic glaciation amplified the climatic effects of orbital forcing.

Geological events are able to modify climate and environment at all the different scales, from a global perspective to strictly delimited areas, thus affecting and driving evolution on Earth. A main process that changes climate is Plate Tectonics: the wandering continents change their latitude, thus modifying the strength of solar radiation reaching their surface; the consequent climate and environment changes constitute major evolutionary forcing.

Other geological factors are orography, sea level, ocean, and landmasses distribution and their variations. Also marine currents and atmospheric winds regime are strongly influenced by landmass and ocean distribution.

The effects of the variation in landmasses distribution can be felt over long distances if they affect ocean currents. For example, in late Tertiary the northward drift of the Australian continent closed the seaway across which the warm waters from Central Pacific went to the Indian Ocean. In consequence the Indian Ocean cooled, and the East African region experienced a second drop in rain rate, after the one occurring when the mountains of the East African Rift rose [1].

The arrival of landmasses in polar area has strong consequences in their climate but also carries on global consequences: the development of ice caps drops sea level, while ice and permafrost retain variable amount of greenhouse gas like CO_2_ and CH_4_, triggering global temperatures falls.

After the break-up of a continent, new clades can rise from the division of formerly single populations, while the union of two continents results in a complex pattern of biotic interchanges, extinctions, and fast and spectacular radiations and differentiations.

Landmass union and intracontinental rifts have raised mountain belts that changed climate and created barriers for rains and biotic movements between the two sides of the uplifted belt.

## 2. Atmospheric CO_**2**_ Amount and* Large Igneous Provinces*

The heavy links between life and continental drift have been well established in the second half of XX century (mainly in the 60s) while the effects of greenhouse changes (particularly CH_4_ and, more, CO_2_) have been poorly underscored until today, but global climate (and above all temperatures and what happens when they change) is a direct consequence of the amount of atmospheric CO_2_, this value being influenced by many factors.

Among atmospheric factors, a main role is played by the greenhouse gas amount. Early Earth atmosphere was composed of more than 95% of CO_2_, as the current ones of Venus and Mars. Young Sun radiation was weaker than in Phanerozoic times: only thanks to these high atmospheric pressure and greenhouse conditions Earth could retain liquid waters in Archean and Paleoproterozoic times, while Earth with the current atmosphere would be globally iced.

Volcanoes emit several hundreds of thousands of tons of CO_2_ yearly: this “ordinary” gas flow from solid Earth to atmosphere is lower than the carbon dioxide demand of the Earth system; thus, currently, the residence time in the atmosphere of a carbon dioxide molecule is shorter than 4 years.

The best-known CO_2_ users are plants and photosynthesis changed the world: more than 2 billion years ago, soon after the onset of this process, atmospheric carbon dioxide content dropped while the rise of oxygen occurred (the* Great Oxygenation Event*) because of photosynthetic unicellular algae; so these tiny organisms changed the environment from reducing to oxidizing conditions and also global temperature got down and the first global glaciation event occurred.

Sea water adsorbs CO_2_ (the more its amount, the more the oceanic acidity); land surface and marine bottom use CO_2_ in silicates minerals weathering and carbonates formations; another strong mineral sequestration process is the formation of hydrocarbons and coals reserves. Other CO_2_ is trapped in marine sediments, returning in the mantle when their plate arrives at a subduction zone.

An important remover of atmospheric CO_2_ is represented by polar areas: the arrival of landmasses in high latitude has strong consequences in their climate but also carries on global consequences: not only does the development of ice caps drop sea level, but, more, ice and permafrost retain huge amount of greenhouse gas like CO_2_ and CH_4_, triggering global temperatures falls. Atmospheric CO_2_ dramatically dropped at the onset of Antarctic glaciation in Neogene and the current occurrence of ice caps is the main reason for the current very low CO_2_ atmospheric content.

Only strong emissions from inner Earth can increase CO_2_ atmospheric amount: this happens during the* Large Igneous Provinces* activity.

*Large Igneous Province* (from here LIP) is a concept introduced by Coffin and Eldhom in 1991 [4], joining into single definition continental* flood basalts* and oceanic plateaus. A LIP is an emplacement of hundreds of thousands of cubic kilometers (also millions of cubic kilometers) of mainly basaltic magmas in a very short period of time: flow-by-flow analysis of paleomagnetic directions along 10 traverses of the Western Ghats in India suggests that at the final Cretaceous a large fraction of the main sequence of the Deccan Traps was formed in a succession of less than 30 short eruptive events in a time interval lasting for less than 10 ka [5]. Similar considerations come from studies performed on Jurassic Karoo Basalts [6] and latest Permian Siberian Traps [7].

**Figure 1:**
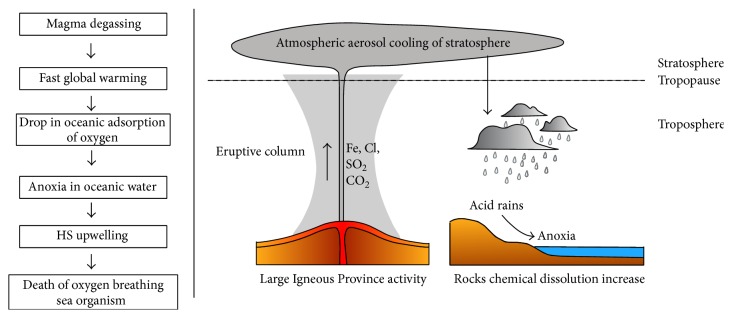
The effects on soil, atmosphere, oceanic waters, and rains of volatiles emitted by a Large Igneous Province.

The emplacement of a LIP often results in a lot of environmental consequences at global scale, affecting atmosphere, soils, and hydrosphere because of the massive magma and volatiles production. This has been well established in Deccan Traps, the LIP that occurred at the end of Mesozoic, clearly related to dinosaur extinction (for a good examination of the topic see a recent volume of the Geological Society of America Special Publications [8]): single eruptive events of a LIP activity produce a lava volume ranging from 1,000 to 20,000 km^3^ in few tens of years; during each of these short-lasting paroxysmal phases the releases of volatiles exceed the sequestration potential of Earth system: in consequence the accumulation in atmosphere of CO_2_, SO_2_, heavy metals, and other substances promotes continuous and strong environmental variations; in this way many parameters increase, like terrestrial weathering patterns, ocean and atmospheric temperatures, and water acidity in the seas and rains; many extinction events are also accompanied by severe sea level oscillations (particularly, a fast transgression preceded by a drop); in the worst cases also anoxic events with a worldwide distribution can occur.  Figure 1 summarizes the climatic effects of LIP emissions in atmosphere.

Laki eruption in 1783 in Iceland can be a pale example of what happens during a LIP flow: in the island volatiles killed most of livestock and a strong famine occurred; in all Europe a sulphur cloud damaged crops and the winter 1784/84 recorded the worst mortality level in England and northern Europe of the century, especially for lung and heart diseases. And Laki produced only 16 cubic kilometers of lava in 8 months, a little quantity when compared with a single event of a LIP activity.

Environmental perturbations related to a LIP emplacement begin well before the spike of volcanic activity, probably because in the early stages the lava produces a huge amount of pyroclastics and volatiles coming from metasomatism and other reactions of the sediments in which they intrude; the higher the thickness and/or the evaporitic content of the sediments, the higher the production [9].

**Figure 2:**
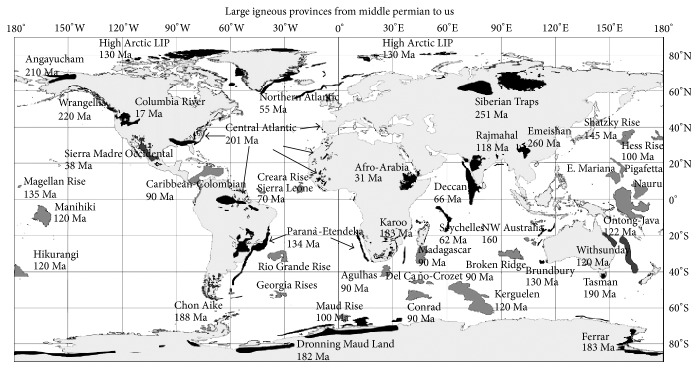
From [2], map of the Large Igneous Provinces emplaced from the Middle Permian (260 million years ago).

Figure 2, from [2], depicts the major LIPs events that occurred from early upper Permian (260 million years ago) until today. There is a clear coupling between LIP emplacements and biosphere changes, the strongest changes being the main mass extinction events. The very high speciation rate immediately before and after an extinction event reflects the continuous adaptation of the biosphere to new conditions: this is demonstrated by the biozones, which follow one another at a faster pace before and after the peak of the crisis, even if diversity generally falls because the extinction rate exceeds by far origination rate.

It is also noticeable that from the end of the Devonian to the beginning of the Eocene all the major chronological divisions in geological times are coeval with the occurrence of a LIP.

**Figure 3:**
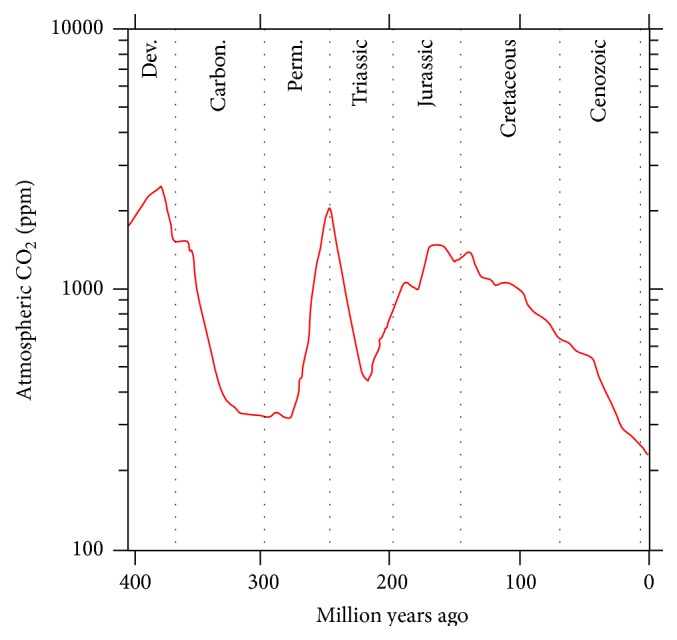
Atmospheric CO_2_ in the last 400 Ma from [3].

Figure 3, modified after [3], depicts the medium atmospheric CO_2_ content from the Devonian to today, calculated analyzing carbon isotope composition from stomatal anatomy of fossil leaves: this method is based on the inverse relationship observed in many plants between stomatal number and CO_2_. CO_2_ was low (<500 ppm) during periods of long-lived and widespread continental glaciations and high (>1000 ppm) during warmer periods [10].

The continuous drop in CO_2_ atmospheric content in the Devonian can be related to plant colonization of the interior of continents [11]: if, during the Early to Middle Devonian, land plants were clustered in lower floodplain and coastal delta habitats, from the Middle to Late Devonian, they began to occupy more upland regions, thus increasing CO_2_ consumption.

This trend was briefly interrupted in the Late Devonian because of the occurrence of some LIPs that injected in the atmosphere a great amount of this gas. A second process concurring with the atmospheric CO_2_ drop started at the beginning of the Permo-Carboniferous glaciation: the arrival of landmasses in both the high latitude areas, Gondwana in the southern hemisphere and Siberia in the northern.

CO_2_ atmospheric content grew at the end of the Permian abruptly, because of the final melting of the Permo-Carboniferous ice caps and because of the very high emissions produced by the Emeishan and Western Siberian Traps: for these reasons and for the occurrence of a large supercontinent in which moisture transport was difficult, the Early Triassic has been very hot and dry [12].

The high temperatures and CO_2_ amount of Mesozoic atmosphere were a direct consequence of the widespread LIP magmatism of that time, related to the onset of the Gondwana break-up. So, LIP magmatism is the main dispenser of this gas and when the frequency of this activity dropped, also atmospheric CO_2_ content did so. In Tertiary LIP activity has been weaker than in the Cretaceous (there have been only 3 smaller events on land and no one oceanic plateau has formed); contemporarily the Antarctic ice cap formation added a new, strong CO_2_ sequestration process, witnessed by the increase in the CO_2_ drop rate from the end of the Oligocene to today. In consequence, CO_2_ amount collapsed, as temperatures did, with plenty of environmental changes worldwide.

## 3. Mass Extinctions and Plants Evolution in the Fossil Record

Life on Earth has been largely affected by various mass extinction episodes, defined by Jack Sepkoski as short-lasting phases showing significant extinction rate in various nonrelated clades at a global scale [13]. Before and after these events life is very different since former dominant groups vanished and new groups, with a rapid origination, assume the leadership.

In 1983 Niklas et al. described four main phases of plant biodiversity growth [14]:

1. The first origination of early terrestrial plants and the rise of the first forest in Silurian times.
2. Ferns and lycophytes of the lower Carboniferous.
3. Gymnosperm diffusion from Upper Carboniferous.
4. The rise and diffusion of the angiosperms from the lower Cretaceous.

A new method,* PyRate*, uses a Bayesian analytical framework to origination and extinction dynamics using fossil occurrence data of plant fossil occurrences from the Paleobiology Database (PBDB; https://paleobiodb.org/cgi-bin/bridge.pl) [15]. Compared to other approaches, this method has the advantage of using all fossil occurrences in the analysis (e.g., not only first and last appearances) and of jointly estimating (1) the preservation rate, (2) times of origination and extinction of all lineages, and (3) rates of origination and extinction through time. The inference is carried out in a sound statistical framework, which has been shown to be robust under many evolutionary and sampling scenarios through extensive simulations [16]. By exploiting the properties of Bayesian modeling,* PyRate* generates parameter estimates provided with credible intervals while accounting for different sources of data and parameter uncertainties.

**Figure 4:**
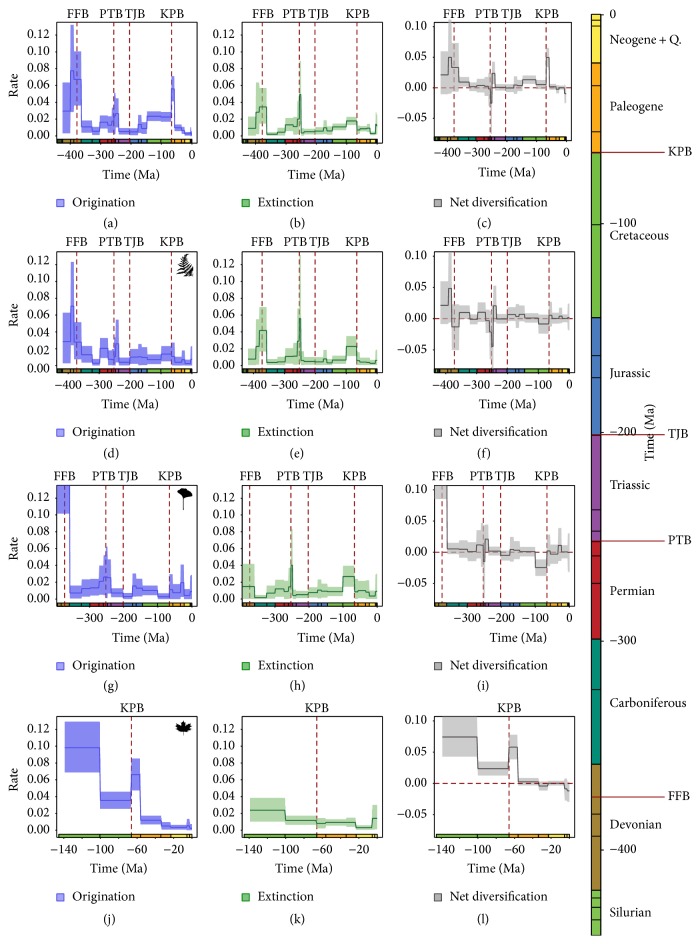
From [17], generic level diversification analysis of all vascular plants. Origination (blue) and extinction (green) rates were estimated using the Bayesian approach implemented in* PyRate*, within time bins defined as epochs of the geologic timescale (shown at the bottom). The timescale (*x*-axis) is given in million years ago (Ma). Solid lines indicate mean posterior rates, whereas the shaded areas show 95% HPD intervals. The diversification dynamics were estimated for vascular plants as a whole (a–c), spore-bearing plants (d–f), nonflowering seed plants (g–i), and flowering seed plants (angiosperms: j–l). Net diversification rates (gray) are defined as origination minus extinction. The dashed lines indicate the major mass extinction events widely recognized: at the Frasnian-Famennian (FFB), Permian-Triassic (PTB), Triassic-Jurassic (TJB), and Cretaceous-Paleogene (KPB) boundaries. Plant silhouettes were obtained from http://phylopic.org/.

*PyRate* tracks vascular plant origin and diversification from the Silurian to today, dividing them into four main clades: vascular plants, spore-bearing plants, nonflowering seed plants, and flowering seed plants. The obtained data are depicted in Figure 4, from [17].

The oldest uncontroversial plant fossils are cryptospores of mid-Ordovician age [18]. At these times CO_2_ atmospheric content was very high, some 8–20 times the preindustrial atmospheric level [19]. Even if climate was not so hot as expected, high CO_2_ levels are undoubtedly a good chance for plants growing, especially with concurrent humid conditions.

## 4. Lower Devonian Expansion of Vascular Plants and the Upper Devonian Biotic Crisis

The first vascular plants lived as early as the Early Silurian.* PyRate* shows a very high vascular plants origination rate in the Devonian, reaching the higher level ever in the first half of the Upper Devonian. This high level is an effect of the radiation of terrestrial plants during a huge increase in biomass amount that strongly changed continental grounds and modified hydrological cycle [20].

Nonflowering seeds plants originated in the Upper Devonian, exhibiting a high origination rate: the development of heterospory in progymnosperms with the spores transformed in seeds allowed them to reproduce also in harsher areas.

This novel reproduction system has been very useful in Devonian continents where arid or semiarid environment was widespread, raising the ability of plant to colonize larger areas than before: the fast radiation triggered a fast evolution and both origination and extinction rates have been constant up to the beginning of the Late Devonian.

In latest Devonian origination rate dropped two times, slightly before the Frasnian-Famennian border (from here FFB) and at the end of the Devonian, this second drop being huger than the first:* PyRate* shows an extinction rate spike some before the FFB, remaining constant throughout the border at slightly lesser levels and exhibiting a dramatic increase in the Famennian.

The Upper Devonian has been a very hard time: plant diversity modifications mirror two main faunal mass extinction events that occurred at the Frasnian-Famennian border (the* Kellwasser event*) and, soon after, at the Devonian-Carboniferous border (*Hangenberg event*). The end of Frasnian and the beginning of Famennian are characterized by fast climate changes, sea level oscillation, excursions in carbon and oxygen isotopic ratios, global anoxia, spike of atmospheric sulfide, and other phenomena.

This biotic event is one of the main Phanerozoic extinctions affecting animals, with a huge drop in biodiversity.* PyRate* demonstrates that also plants suffered a lot that time. Like most of mass extinctions, these events have been driven by the emplacement of Large Igneous Provinces: the Frasnian-Famennian border is clearly linked with the* Viluy Traps* in Yakutia [21]. The end Frasnian biotic crisis was followed by a second one at the very end of the period with widespread anoxic sediments and perturbation of the carbon isotopic ratio. Geological and geochemical observations witness that also the* Hangenberg* event is closely related to the emplacement of a LIP, but the position of the volcanic center has not been currently determined, Ogcheon Belt in Korea being the most candidate site today [22]. It is noticeable that the LIP activity briefly interrupted the dropping trend of atmospheric CO_2_ content.* PyRate* demonstrates that the geological events of the Upper Devonian have played a major role in plants evolution, since their diversity at the beginning of the Carboniferous was well lower than at the onset of the Upper Devonian storm.

## 5. Carboniferous Plants: A Two-Faced History

In the lower Carboniferous climate has been warm, thanks to the high CO_2_ emissions from Late Devonian LIPs, and it was also very wet in the equatorial belt, where the main plant biomass was hosted. The climate was also stable that time, so, despite the floral abundance, either origination or extinction rate was slow.

We must also note a dramatic fall of atmospheric CO_2_ throughout all the lower Carboniferous [23]. These dryer conditions have been harder for spore-bearing and favored nonflowering seed plants. This extinction event is not related to a LIP occurrence, as occurred well later, at the Eocene-Oligocene border: it is interesting that both these biotic crises are placed near the beginning of the two last Phanerozoic major glaciation phases.

So, we can say that mid-Carboniferous rainforest collapse witnesses another main link between geology and plant evolution, even if its exact causal mechanism is still poorly known. Climate becomes colder and dryer than before throughout the Upper Carboniferous, with very low atmospheric content of CO_2_ and methane, also because they have been partly trapped in the large areas covered by thick ice caps and permafrost and in high latitude seafloor.

Whatever the geological origin of the problems affecting vascular plant in the mid-Carboniferous, in good agreement with the drying climatic trend,* PyRate* detects opposite behaviors in spore-bearing and nonflowering seed plants diversity between the lower and Upper Carboniferous: in the Upper Carboniferous spore-bearing plants reduced their diversity, with a huge drop in speciation and a very little extinction rate compared to the first half of the period, while nonflowering seeds plant performed a diversity growth even if they have been affected by a stronger extinction rate than before.

It is interesting that in Upper Carboniferous reptiles performed a wide radiation that, like the Devonian nonflowering seed plants diversification, has been driven by a reproductive step: the new eggs allowed the birth of an animal quite similar to an adult with very fewer needs of water than an aquatic tadpole.

## 6. Permian and the End-Permian Global Crisis

Also in the Permian the lower and the upper part of the period show different trends in plant diversity, related to different climate conditions: the Early Permian marks the end of the major high latitude Gondwana glaciation [24]. The dropping CO_2_ trend stopped. Thanks to a global temperature and moisture rise a huge increase in spore-bearing plants diversity occurred in the lower Permian, well detected by* PyRate*.

The warming trend can be partly ascribed to the earliest Permian* Skagerrak basalt* LIP emissions. The end of the Permian as well as the beginning of the Triassic has been one of the most difficult times for life on Earth: two main faunal extinction events occurred, one near the end of the Capitanian and the second at the end of the period, less than 8 Ma later. The faunal replacement has been so heavy that the end of the Permian has been the worst extinction event ever, establishing the limit between Paleozoic and Mesozoic era since the half of XIX century.

These events have been caused by the emplacement of two Large Igneous Provinces: the* Emeishan Traps*, now Southern China, and the* Siberian Traps*, east of the Ural Mountains.

Plants have been severally affected by the extinction: greenhouse gas and volatiles raised temperature and rain acidity in a drying world [25], triggering massive environmental changes that drove, either in spore-bearing plants or in nonflowering seed plants, the most dramatic diversity drop ever. But the diversity behavior shows a complex trend.

Spore-bearing plants slowed their origination rate in early upper Permian times: the onset of volcanic activity in Southern China coincided with their fall in origination rate and increase in extinction rate that began well before the faunal extinction spike of the upper Capitanian. In the latest Permian, between the two faunal extinction spikes, a lower origination rate is accompanied by a dramatic increase in extinction, prolonged toward the earliest Triassic, when it has reached its maximum level. Nonflowering seed plants show a similar pattern.

So,* PyRate* suggests heavy links between plant history and the environmental storm triggered by the volatiles released during the emplacement of Emeishan and Siberian basalts. It is noticeable that a spike in origination rate occurred after the second LIP activity in the earliest Triassic, but it could not compensate the even major extinction rate: thanks to the very high level of CO_2_, partly of deglaciation origin, partly emitted during both LIPs' activity, these times have been the hottest and the driest in the Phanerozoic Eon [11], a very hostile period for life. The high plants origination and extinction rates demonstrate that environmental changes have been strong, frequent, and continuous during all the latest Permian and earliest Triassic.

## 7. Mesozoic Era and the Rise of Flowering Seed Plants

After the first 6 Ma of the Triassic, temperatures went down, thanks to the various CO_2_ sequestration processes, allowing plants and animals to reconquer the equatorial belt in the early Anisian. In this stage diversity fast increased either in spore-bearing or in nonflowering seed plants. During the following part of the Triassic there are no major changes in plant biodiversity patterns.

The Triassic ends with the emplacement of the* Central Atlantic Magmatic Province*, the initial stage of Pangea break-up; magmatic activity triggered a severe mass extinction event in animals: many clades of Archosaurs got extinct, as most of mammalian reptiles, and a fast dinosaurs radiation and differentiation took place. This event did not affect vascular plants.

Early Jurassic warm and wet environment promoted a high differentiation in spore-bearing plants. A peak in speciation and extinction is placed in the middle Jurassic, with a low drop in diversity of spore-bearing plants while diversity in nonflowering seed plants little increased. This scenario can be related to a minor extinction event at the end of the Toarcian, corresponding to a global anoxic stage, clearly related to the environmental effects of the* Karoo-Ferrar* LIP, immediately before the beginning of the Gondwana break-up: a little later the blocks formed by Africa-Arabia and South America split out from the block formed by India, Antarctica, and Australia.

In Cretaceous times the main event for vascular plants has been the strong angiosperms diversification.

The origin of this group is debated because molecular data point to an older, Triassic age for this clade, when the ancient most fossil has been found in lower Cretaceous sediments, even if there are controversial claims about some Triassic fossils.

Thus, there is a gap of several tens of millions of years between the molecular and the paleontological timing (no less than 60). Many explanations have been proposed for this bias: it could be simply of paleontological origin (the lack of fossils) but also methodological issues in the analysis of genetic data must be considered: for a good and recent examination of the topic see Beaulieu et al. (2015) and references therein [26].

Could the rise of angiosperms be well older than their huge radiation?

For Doyle Triassic molecular dates may be reconciled with the fossil record if the first angiosperms were restricted to wet forest understory habitats and did not radiate outside this environment until the Cretaceous [27].

If this last reconstruction is true, something has occurred promoting the nonflowering seed plants replacement with angiosperms.

Also, in this case, a geological trigger can be considered. Climate was hot thanks to the high, even if lower than in the early Triassic, CO_2_ content that grew (up to four times higher than today), because of the widespread LIPs activity of the lower Cretaceous, during which the Caribbean, Ontong Java-Manihiki-Hikurangi, Madagascar, Kerguelen, and other more Large Igneous Provinces had been emplaced. Climate was wetter than the early Triassic, because of the completely different arrangement of the continents: Pangaea break-up resulted in newborn oceans interposed between smaller landmasses compared to the former supercontinent; these oceanic arms allowed humidity distribution in larger areas than before, diminishing the extension of arid and semiarid lands.

Moreover, most of the boreal continent Laurasia was placed at low northern latitudes, in the tropical area.

Thanks to both of these concurrent factors, higher CO_2_ levels and wider humidity distribution, plant biomass was higher than before, resulting in an increase of photosynthetic activity: in this way also atmospheric oxygen increased. The higher atmospheric oxygen content raised the occurrence of wildfires that became very common later, in the upper Cretaceous, witnessed by the widespread occurrence of fullerenes in the sediments. Because of their resins content, wildfires can have represented a major challenge for conifers more than for angiosperms that replaced nonflowering seed plants in many environments [28].

In the upper Cretaceous,* PyRate* shows a drop in all vascular plants, also in angiosperms.

This is apparently strange, because the Gondwana break-up divided into some blocks the former greater continent and this event theoretically would have triggered a diversity increase.

Why is this decline in a commonly reputed happy period for life and of splitting continents? Climate becomes newly harsher, with pronounced seasonality also at low latitudes, and an aridification of waters, rains, and soils occurred, while the high atmospheric O_2_ and the prolonging of dry seasons increased wildfires frequency, dramatically affecting plants diversity. Also various episodes of sea level oscillation can be blamed for this diversity drop which occurred also in animals.

The Cretaceous ended with the most famous of all mass extinctions, in which not only dinosaurs have been wiped off. As written before, for this event the Yucatan meteorite impact has been blamed but this hypothesis has been challenged, and now another LIP, the Deccan Traps, is currently the best candidate for the killer role [29], because the chain of events recorded in latest Cretaceous sediments is the typical sequence of the geological, geochemical, and environmental disturbance related to a LIP emplacement.

## 8. From the Paleocene to Today: Vascular Plants Diversity in the Global Cooling Trend

As what happened in the other biotic restorations before mass extinctions, the Paleocene is characterized by a huge origination trend in all vascular plants, in the aftermath of K/T extinctions and environmental disturbance. From the Eocene to today, origination rates in nonflowering seeds plants and fern-bearing plants show trend opposite to that of the flowering plants: being higher in the Oligocene and Quaternary for the former, while the latter shows higher trends in the Eocene and Pliocene: crossing climatological data with* PyRate*-inferred plant diversity, in the Tertiary the warmer the period, the higher the origination rate in flowering plants, while colder periods show an increase in nonflowering and spore-bearing plants.

After the climax in the middle of the late Cretaceous, global temperatures began to fall: the Mesozoic greenhouse climate has been driven by the widespread occurrence of LIPs; but since their frequency lowered, Earth system has been able to soak up much more CO_2_ than the ordinary volcanic emission amount: in consequence also temperatures trended downward after the highest point reached during the Turonian, 30 Ma before the end of the Cretaceous (90 Ma).

However Eocene climate has been still warm, accompanied by two thermal highs, at its beginning (PETM (*Paleocene Eocene Thermal Maximum*), synchronous with the main phase of the North Atlantic Igneous Province) and in the middle (MECO (*middle Eocene climatic optimum*)). Rainforest was widespread also in the areas now covered by deserts or savannas of Africa, Arabia, and SW Asia at time. But at the Eocene-Oligocene border the ongoing cooling trend triggered also a little faunal extinction event (*Mongolian Remodelling* or* Grand Coupure*).

There is probably a link between Eocene-Oligocene border cooling,* Mongolian Remodelling*, and the onset of Antarctic glaciation, an event for which many forcings acted:

- The global scale atmospheric CO_2_ continuous fall.
- The arrival of Antarctica near south pole.
- The split between Antarctica and Australia and the Drake Strait opening that allowed the circumpolar current formation, preventing since then the mixing of polar and warmer waters in southern ocean [30].

From that moment permafrost and ice caps began their development in southern hemisphere (in northern hemisphere their onset is later, since North America and Eurasia arrived later than Antarctica at their current high latitudes), hence becoming a new, main, greenhouse gas sequestration process. From the beginning of the Oligocene, rainforest began to fall in higher latitudes, replaced by an open woodland environment also in Southern Africa.

It is noticeable also that these geological changes promoted in many areas C3 plants replacements with C4 plants.

## 9. Conclusions

Vascular plants diversity dropped during various mass extinction events, especially at the two Upper Devonian events, Permian-Triassic border, Toarcian and Cretaceous-Paleogene border, while they passed the Triassic-Jurassic border event without problems. Diversity pattern shows a growth before extinction peaks, a common behavior in the frame of the biosphere recovery after a mass extinction.

Plant recovery has been slightly delayed only after the End-Permian extinction, because of the extreme climatic conditions created by the double LIP event (Emeishan Traps, Permian Traps) and by the final sudden vanishing of the last Permo-Carboniferous ice caps.

The data inferred with* PyRate* are in excellent agreement with geological events, thus suggesting a heavy link between plant evolution and geological events, especially the occurrence of Large Igneous Provinces.

So, the four stages detected by Niklas et al. in 1983 [14] can be interpreted with environmental changes related to geological events:

1. The high CO_2_ level allowed the origination of early terrestrial plants and the rise of the first forest in Silurian times.
2. The wide occurrence in the lower Carboniferous of lands along the equatorial belt allowed the rise of the tropical rainforest and the diffusion of ferns and lycophytes.
3. The arrival of large landmasses in polar positions and lowering in LIP occurrence rate originated the cooling and aridification trends of the Upper Carboniferous culminated with the onset of the Permo-Carboniferous glaciation, thus promoting the nonflowering seed plants diffusion.
4. The diffusion of the angiosperms from the lower Cretaceous has been a consequence of the wide wildfires occurrence, triggered by the climatic and environmental consequences of the massive volcanic activity of that time and by the Pangaea break-up in a world in which moisture was more widely distributed than before.
